# Outstanding Reviewers for *Nanoscale Advances* in 2022

**DOI:** 10.1039/d3na90060f

**Published:** 2023-06-23

## Abstract

We would like to take this opportunity to highlight the Outstanding Reviewers for *Nanoscale Advances* in 2022, as selected by the editorial team for their significant contribution to the journal.
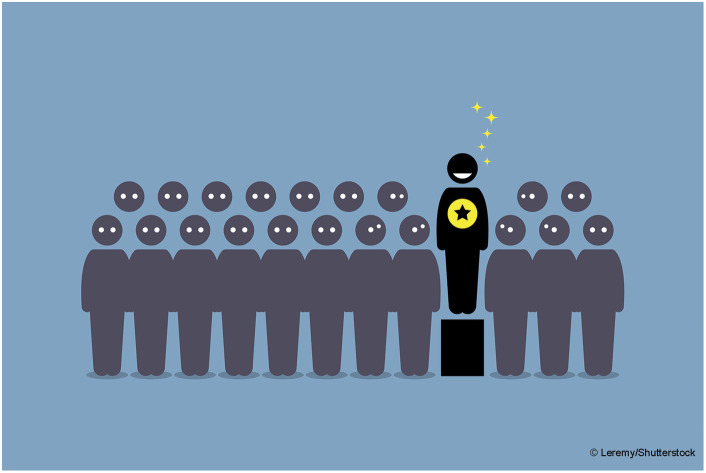

We would like to take this opportunity to thank all of *Nanoscale Advances’* reviewers for helping to preserve quality and integrity in chemical science literature.

We would also like to highlight the Outstanding Reviewers for *Nanoscale Advances* in 2022. Each one of our outstanding peer reviewers has been carefully selected by our editorial team and includes active researchers who have made significant contributions to peer review and have gone above and beyond in their actions.

“*We are delighted to recognise Nanoscale Advances Outstanding Reviewers. Guaranteeing the quality and impact of Nanoscale Advances is only made possible through a stringent peer review process, with two stand-out aspects: on the one hand, excellence of the reviews, and, on the other hand, timeliness. At the heart of peer review is carefully drafted reports; reports that provide a valuable service to the scientific community and to the readers of Nanoscale Advances. On this occasion, I want to extend a big thank you to these Outstanding Reviewers and everyone else who has reviewed manuscripts for Nanoscale Advances.*” – Professor Dirk Guldi, Editor-in-Chief

Dr Peng Huang

Shenzhen University

ORCID: 0000-0003-3651-7813

Dr Hiang Kwee Lee

Nanyang Technological University

ORCID: 0000-0003-0823-4111

Dr Bo Li

Kennesaw State University

ORCID: 0000-0001-9407-9503

Dr Ali Makky

University Paris-Saclay

ORCID: 0000-0002-3807-0886

Dr Christopher Jay Torres Robidillo

University of the Philipines Manila

ORCID: 0000-0001-6492-5107

Dr Chaoliang Tan

City University of Hong Kong

ORCID: 0000-0003-1695-5285

Professor Dr Mengye Wang

Sun Yat-Sen University

ORCID: 0000-0002-0701-5948

Dr Ershuai Zhang

Wayne State University

ORCID: 0000-0001-6866-2762

Professor Dr Tierui Zhang

Technical Institute of Physics and Chemistry, Chinese Academy of Sciences

ORCID: 0000-0002-7948-9413

We would also like to thank the *Nanoscale Advances* Editorial Board and Advisory Board and the nanoscience community for their continued support of the journal, as authors, reviewers and readers.

We continue to work on improving the diversity of our reviewer pool to reflect the diversity of the communities that we serve.

Chunli Bai, Editor-in-chief

Dirk Guldi, Editor-in-chief

Jeremy Allen, Executive Editor

## Supplementary Material

